# Rheumatoid Factor: Diagnostic and Prognostic Performance and Therapeutic Implications in Rheumatoid Arthritis

**DOI:** 10.3390/jcm14051529

**Published:** 2025-02-25

**Authors:** Tasuku Togashi, Ryuhei Ishihara, Ryu Watanabe, Mayu Shiomi, Yuya Yano, Yuhei Fujisawa, Masao Katsushima, Kazuo Fukumoto, Shinsuke Yamada, Motomu Hashimoto

**Affiliations:** Department of Clinical Immunology, Osaka Metropolitan University Graduate School of Medicine, Osaka 545-8585, Japan

**Keywords:** anti-citrullinated protein antibodies, rheumatoid arthritis, rheumatoid factor, tumor necrosis factor

## Abstract

Rheumatoid factor (RF) is the first autoantibody identified in rheumatoid arthritis (RA) which targets the fragment crystallizable (Fc) region of immunoglobulin (Ig) G. Although IgM isotype is predominant, other Ig isotypes, including IgG and IgA, also exist. While RF is not specific to RA, it remains a valuable serological test for diagnosing the disease, as evidenced by its inclusion in the 2010 classification criteria for RA based on elevated serum RF levels. RF is also associated with RA severity, including joint damage and extra-articular manifestations, serving as a poor prognostic factor and aiding in the identification of difficult-to-treat RA. Recent studies have demonstrated that high serum RF levels are associated with a reduced response to tumor necrosis factor (TNF) inhibitors. In contrast, anti-TNF antibodies lacking the Fc portion have shown stable efficacy in RA patients regardless of baseline RF levels. These findings reaffirm the clinical significance of RF measurement, 80 years after its initial discovery. This review explores the diagnostic and prognostic significance of RF and its impact on treatment selection in RA management.

## 1. Introduction

**Figure 1:**
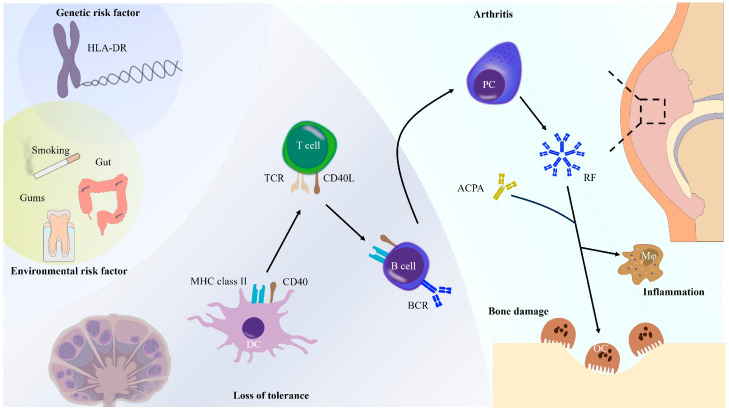
RF implicated in the pathogenesis of RA. The onset of rheumatoid arthritis (RA) is driven by a complex interplay of genetic and environmental factors. These factors lead to the generation of exogenous and endogenous antigens, which are subsequently presented by antigen-presenting cells (primarily dendritic cells) to CD4+ T cells. Upon activation, CD4+ T cells differentiate into self-antigen-specific subsets, including follicular helper T cells (Tfh) and peripheral helper T cells (Tph). These specialized CD4+ T cells facilitate B cell maturation and promote their differentiation into memory B cells and long-lived plasma cells. Plasma cells in inflamed synovial tissue produce significant quantities of rheumatoid factors (RF), predominantly Immunoglobulin M (IgM) type, and anti-citrullinated protein antibodies (ACPA). These autoantibodies form immune complexes with self-IgG in the synovium of RA patients. These immune complexes amplify complement activation, cytokine production by macrophages, and exacerbate joint damage by promoting osteoclast activation. Abbreviations: DC, dendritic cell; PC, plasma cells; Mφ, macrophage; OC, osteoclast; MHC, Major Histocompatibility Complex; TCR, T-cell receptor; BCR, B-cell receptor.

Rheumatoid arthritis (RA) is a systemic autoimmune disease characterized by chronic, progressive inflammation of synovial joints, often leading to irreversible structural damage to bone and cartilage [1]. This debilitating condition significantly impacts patients’ quality of life and imposes a considerable economic burden on healthcare systems worldwide. Despite extensive research, the precise etiology of RA remains elusive. Several hypotheses, including genetic predisposition, environmental triggers, and dysregulated immune responses, have been proposed (Figure 1); however, none provide a comprehensive explanation for the disease’s onset. Notably, evidence of arthritis-like changes has been identified in skeletal remains from prehistoric times, suggesting that RA is not a modern ailment but rather a condition that has coexisted with humanity for millennia [2].

Epidemiologically, RA affects approximately 0.46% of the global population, as indicated by a recent meta-analysis (95% confidence interval 0.39–0.54) [3]. This prevalence underscores the necessity for rheumatologists and general physicians alike to possess a robust understanding of the disease. Given its chronic and systemic nature, early and accurate diagnosis of RA is critical to initiating timely interventions that can mitigate disease progression and improve outcomes.

The diagnosis of RA is guided by several established classification criteria, among which the American College of Rheumatology/European League Against Rheumatism (ACR/EULAR) 2010 criteria are most widely used in clinical practice [4]. These criteria integrate clinical, serological, and imaging parameters to enhance diagnostic accuracy. In recent years, beyond these classification frameworks, there has been an increasing emphasis on early diagnosis and the incorporation of advanced imaging modalities, such as ultrasound and magnetic resonance imaging (MRI), which are pivotal in detecting subclinical synovitis [5,6].

Among the serological markers, rheumatoid factor (RF) and anti-citrullinated protein antibodies (ACPA) hold particular prominence. RF, a classical autoantibody discovered over eight decades ago, has been a cornerstone of RA diagnostics and research. Although its utility has been debated due to its presence in other autoimmune and inflammatory conditions, recent advancements have shed new light on its role, mechanisms, and potential implications in the pathophysiology and treatment of RA.

The evolving understanding of RF extends beyond its traditional use as a diagnostic tool. Emerging evidence suggests that RF plays a contributory role in disease mechanisms, including the formation of immune complexes and activation of complement pathways, which exacerbate joint damage. Furthermore, the prognostic value of RF in predicting disease severity and therapeutic response has gained increasing attention.

This review aims to provide a comprehensive exploration of the current evidence surrounding RF, with a focus on its clinical and biological significance in RA. We will discuss its historical context, diagnostic and prognostic performance, and therapeutic implications. By synthesizing recent advancements, we hope to elucidate the multifaceted roles of RF in RA and highlight potential directions for future research in this field.

## 2. Diagnostic Performance of RF

### 2.1. Detection Methods and Isotypes of RF

RF encompasses autoantibodies that recognize the fragment crystallizable region of immunoglobulin G (IgG) and were the first biomarkers identified in RA [7]. The discovery of RF dates back to 1940 when Waaler described a hemagglutinating factor in the serum of patients with RA [8]. Subsequently, in 1949, Pike coined the term “rheumatoid factor” to reflect its association with RA [9]. Since then, RF has remained a cornerstone in the study of autoimmune diseases, with its detection methods and clinical implications evolving significantly over the decades.

**Figure 2:**
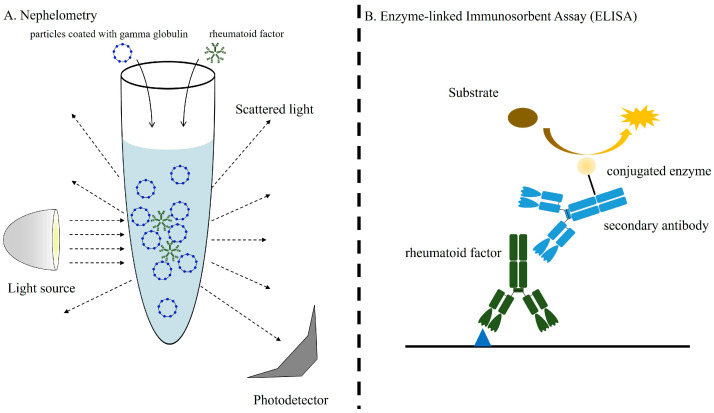
Methods for RF detection. (**A**) Nephelometry is based on the RF-induced aggregation of IgG-coupled particles. A laser beam is directed at the aggregates, and the intensity of the scattered light is quantified. The titer (IU/mL) is calculated from a standard curve, using serum aligned with the WHO’s reference standard. The specific contribution of the various RF isotypes to the aggregation observed by nephelometry remains undetermined. (**B**) In the enzyme-linked immunosorbent assay (ELISA), the Fc portion of purified human IgG is immobilized on polystyrene microtiter plates and reacted with test serum. RF bound to the antigen is detected using an enzyme-conjugated secondary antibody specific to the immunoglobulin class, followed by the addition of an enzyme substrate.

Various techniques have been developed for the measurement of RF, ranging from classical nephelometry and turbidimetry assays to more advanced methodologies. The former are isotype-nonspecific assays (Figure 2A) [10], while the advent of enzyme-linked immunosorbent assays (ELISA), fluorescence enzyme immunoassays (FEIA), chemiluminescence immunoassays (CLIA), and chemiluminescence microparticle immunoassays (CMIA) has allowed for isotype-specific RF detection, significantly advancing our understanding of its clinical relevance (Figure 2B) [11].

The first World Health Organization (WHO) RF standard serum was established by pooling RA sera collected in 1963. In 1964, this pooled serum was divided into three batches, with the first batch designated as the international standard serum, W1066 [12]. The second batch was used to develop the first British standard serum, known as 64/002 [13]. Given their shared origin, W1066 and 64/002 are interchangeable as reference materials for RF measurement. The collaborative study involved 11 laboratories across 7 countries, all of which were required to utilize the sheep cell agglutination assay. Presently, most commercially available RF tests are standardized to W1066, with results expressed in international units (IU)/mL. However, at the time of the WHO standard W1066’s publication in 1970 [14], isotype-specific assays had not yet been implemented. Results for IgA-RF and IgM-RF exhibit significant variability among commercially available assays, rendering them non-interchangeable for clinical and research applications [15,16].

Four subclasses of RF are detected by ELISA: IgM, IgG, IgA, and IgE [17], although IgE-RF measurements are rarely performed in recent years. Each isotype exhibits distinct associations with disease phenotypes and outcomes. IgG-RF and IgE-RF are particularly noteworthy for their links to vasculitic manifestations [18,19]. Additionally, IgE-RF levels are low in approximately half of patients with rheumatoid arthritis, whereas elevated concentrations are observed in individuals with rheumatoid vasculitis and Felty syndrome [19,20]. Conversely, patients positive for IgA-RF or IgM-RF often present with higher disease activity and greater radiological damage compared to seronegative patients [21,22,23,24,25]. Moreover, IgA-RF has been significantly associated with pulmonary lesions, while IgM-RF correlates strongly with the presence of rheumatoid nodules [26].

### 2.2. Sensitivity and Specificity of RF

The sensitivity and specificity of RF in diagnosing RA vary depending on disease stage, RF isotype, and detection method. The sensitivity of IgM-RF for RA is estimated to range from 41% to 66% in early RA and increases to 62% to 87% in established RA [27,28]. These figures underscore the dynamic nature of RF’s diagnostic utility across the disease continuum. Moreover, the diagnostic performance of individual RF isotypes has been the focus of numerous studies, reflecting the complexity and heterogeneity of RF expression in RA patients.

A comprehensive systematic review and meta-analysis, involving 36 peer-reviewed studies and 7517 patients, provided insights into the sensitivity and specificity of various RF detection methods. Among the methods assessed, nephelometry and latex agglutination achieved the highest overall sensitivity (68.6%) across all isotypes. In contrast, isotype-specific assays such as ELISA and FEIA highlighted notable differences in sensitivity: IgM-RF exhibited the highest sensitivity (63.4%), followed by IgA-RF (49.1%) and IgG-RF (39.9%) [29]. Regarding specificity, IgA-RF demonstrated the highest specificity (91.4%), followed by IgM-RF (90%) and IgG-RF (78.9%). These results affirm IgM-RF as a robust marker for RA, with a diagnostic odds ratio of 21.7, compared to 16.0 for IgA-RF and 14.2 for IgG-RF, underscoring its clinical utility in identifying RA cases [29]. Furthermore, the presence of multiple RF isotypes enhances the likelihood of RA, highlighting the potential benefit of combined isotype detection in diagnostic and prognostic assessments [15,30].

Despite its diagnostic value, RF has limitations that complicate its interpretation. RF is positive in 5–25% of healthy individuals, and its prevalence increases with age, exceeding 25% in individuals older than 85 years [31]. Moreover, RF positivity has been reported to be more common among women and older individuals than men in the general population of the United States [32]. Additionally, in certain North American Indian tribes, the prevalence of RF positivity among healthy individuals reaches approximately 30%, highlighting population-specific variations in its distribution [33]. Even in studies focusing exclusively on patients with RA, RF positivity rates in the United States demonstrate racial and ethnic disparities, with lower prevalence reported in white individuals compared to Black, Hispanic, and Asian populations [34]. A more recent meta-analysis further revealed that among white individuals, men were significantly more likely to be seropositive than women. However, no significant association between sex and RF seropositivity was observed in Hispanic or Asian populations [35]. These findings suggest that proposing age- and sex-adjusted cutoff values for RF may hold clinical significance.

Additionally, RF is detected in various non-RA conditions, including other autoimmune diseases (e.g., Sjögren’s syndrome and systemic lupus erythematosus), bacterial infections (e.g., infective endocarditis), viral infections (e.g., hepatitis C virus and Epstein–Barr virus), and cryoglobulinemia [28,36]. Environmental factors such as smoking have also been linked to RF production, independent of genetic predispositions like shared epitope alleles [37]. These findings highlight the importance of careful interpretation of RF results in clinical practice.

### 2.3. Distinct Binding Epitopes of RF in Healthy State and Autoimmunity

The mechanisms underlying RF production remain incompletely understood and appear to vary across conditions. RF can be produced during immunizations and secondary immune responses to infections, aiding in pathogen clearance [38,39,40]. In primary Sjögren’s syndrome, RFs are linked to salivary gland mucosa-associated lymphoid tissue–type lymphomas, exhibiting high affinity and monoreactive binding to the IgG-Fc portion [41]. RFs may contribute to the activation and proliferation of B cells in chronic inflammatory sites via IgG immune complexes, increasing the risk of lymphoma development [41]. According to Wang et al., serum RFs in patients with primary Sjögren’s syndrome exhibit clonal restriction and predominantly comprise a shared set of immunoglobulin M heavy chain variable region (IgVH) subfamilies, including 1–69, 3–15, 3–7, and 3–74. Furthermore, certain RF clonotypes have been detected in serum several years before the onset of mixed cryoglobulinemia (MC), and their levels decline following immunosuppressive therapy and MC remission, suggesting their potential role as early biomarkers and indicators of therapeutic response [41]. Recent studies have revealed significant differences in the RF repertoire between RA and other conditions. For instance, RF binding epitopes in healthy individuals and primary Sjögren’s syndrome differ markedly from those in RA, suggesting distinct functional roles [42]. This distinction emphasizes the need for refined diagnostic assays that can differentiate between pathogenic and non-pathogenic RF.

Recent advancements in next-generation RF assays offer promising avenues for improving diagnostic accuracy [43]. These assays, which target RA-specific epitopes, have demonstrated potential in combination with ACPA to identify individuals at high risk of progressing to RA. Such tools hold promise for early diagnosis and intervention, particularly in preclinical or at-risk populations. Further studies are needed to validate these findings.

### 2.4. Comparison Between RF and ACPA

ACPA represent a more recently identified serological marker compared to RF and specifically target citrulline residues of filaggrin present in epithelial cells [44]. ACPA has been recognized for its early detection in the disease process, turning positive before IgM-RF during the preclinical phase of RA [44,45]. Extensive research has highlighted the critical role of ACPA in RA pathogenesis and its superior diagnostic performance. ACPA exhibits higher sensitivity and specificity than RF, particularly in the early stages of RA [46]. Furthermore, studies have demonstrated that double positivity for both RF and ACPA provides greater specificity than single-marker positivity, reinforcing the importance of simultaneous measurement of these antibodies for accurate diagnosis and disease classification [47].

Recent studies have identified glycosylation in the Fab region of ACPA as a distinctive feature associated with RA, suggesting its potential influence on autoantigen binding and the selection and activation threshold of B cells [48]. Beyond their role in disease progression, ACPAs have also exhibited therapeutic potential. Emerging evidence suggests that certain monoclonal ACPAs can attenuate arthritis and inflammation in murine models, underscoring their complex immunomodulatory properties [49]. Further research is needed to elucidate the precise mechanisms of ACPAs in RA pathogenesis and to explore their potential applications in targeted therapeutic strategies.

## 3. Prognostic Performance of RF

### 3.1. Disease Activity and Joint Destruction

Beyond its diagnostic utility, RF holds significant prognostic value in RA, offering insights into disease progression, severity, and treatment response. Studies consistently demonstrate that RF-positive patients at diagnosis are more likely to experience aggressive disease phenotypes characterized by greater joint damage, elevated inflammatory markers, and increased extra-articular manifestations compared to RF-negative individuals [50,51,52,53,54,55]. Notably, patients with high RF titers exhibit a faster progression to joint destruction, necessitating early and aggressive disease control [52,56,57,58]. These observations, supported by multiple studies, highlight RF’s prognostic role in disease stratification and treatment planning [47,55,59].

It has recently been reported that the inflammation-inducing ability of RF is maximized in the presence of ACPA; IgM-RF forms a pentamer, which recognizes the Fc portion of ACPA and forms an immune complex, which stimulates macrophages and others to produce IL-6, TNFα, IL-1β etc. [60]. The combination of RF positivity with ACPA has been linked to higher rates of radiographic progression and poorer functional outcomes [61,62,63,64,65].

RF isotypes may further enhance its prognostic significance. Most of previous studies showed that among RF-positive patients, IgA-RF and IgM-RF are associated with severe disease courses [21,22,23,24,25]. However, recent meta-analysis demonstrated that the prognostic role of RF isotypes is not uniformly definitive, with inconsistent findings reported across studies [29]. While IgM-RF demonstrated modest associations with disease activity in 60% of cases, and IgA-RF showed predictive value for radiographic progression in approximately 50% of cases, results varied significantly due to differences in study designs and outcome definitions [29].

Conversely, a study has reported that the mean disease activity and quality of life in seronegative RA patients are comparable to those of seropositive RA patients, with similar proportions achieving disease remission [66]. Furthermore, certain findings suggest that seronegative RA patients may exhibit greater inflammatory activity than their seropositive counterparts, as assessed by ultrasound and radiography [67,68]. These observations challenge the conventional perception that seronegative RA represents a milder disease phenotype, leaving the debate regarding its relative severity unresolved.

### 3.2. Disease Progression

RF holds significant potential in predicting the onset of RA. RF levels are known to gradually increase over several years, up to 10 years, prior to the onset or diagnosis of RA [45]. Early studies have demonstrated that approximately 17% of IgM-RF-positive individuals presenting with joint pain eventually develop RA during the disease course [69]. More recently, a prospective cohort study conducted in Denmark reported that healthy individuals with elevated RF levels (>100 IU/mL) faced up to a 26-fold increased risk of developing RA, with a 10-year absolute risk as high as 32%. Even individuals with low-titer RF (25–50 IU/mL and 50.1–100 IU/mL) were found to have a 3.6-fold and 6.0-fold higher risk, respectively, of progressing to RA [70]. Notably, the sequential elevation of RF isotypes—beginning with IgM-RF, followed by IgA-RF and IgG-RF—suggests a temporal progression in isotype expression prior to the emergence of clinical manifestations [45,71,72]. These observations underscore the importance of longitudinal RF isotype monitoring as a tool for identifying individuals at risk of developing RA. Such monitoring could enable the implementation of early intervention strategies. Early detection not only improves diagnostic precision but also facilitates timely therapeutic interventions during the critical “window of opportunity”, thereby potentially altering the disease trajectory [57,73,74,75,76,77].

### 3.3. Extra-Articular Manifestations

RA sometimes exhibit extra-articular manifestations [78,79,80], which are notably more prevalent in seropositive patients and those with a prolonged disease duration [81]. Among them, cardiovascular and pulmonary diseases represent the leading causes of mortality among RA patients [82]. RF assessment is essential to understand these systemic complications.

Previous studies have consistently shown that patients with RA face a significantly increased risk of coronary artery disease, congestive heart failure, and sudden cardiac death compared to the general population [24,83,84,85,86,87,88]. Additionally, Federico et al. reported that the presence of positive autoantibodies, including RF and ACPA, is associated with an elevated risk of future cardiovascular damage in patients with early-stage RA, within the first year of disease onset [89]. Furthermore, another study indicated that RF and antinuclear antibodies (ANA) are linked to an increased risk of cardiac disease and overall mortality, even in individuals without rheumatic disease [90]. Notably, disease activity and severity in RA are strongly correlated with cardiac dysfunction, and treatment for RA have been shown to improve cardiac outcomes [88,91,92,93]. Therefore, effective stratification of high-risk patients may play a crucial role in reducing cardiovascular events through the implementation of optimized immunomodulatory therapies.

Similarly, the presence of RF and ACPA has been demonstrated to correlate with a higher incidence and mortality of RA-associated interstitial lung disease (RA-ILD), a serious complication that carries substantial mortality risk [11,94,95,96,97,98,99,100]. Furthermore, high-titer RF seropositivity (≥60 IU/mL) has been linked to mediastinal lymph node enlargement, CT-detected honeycombing, and reduced transplant-free survival, underscoring its prognostic value in RA-associated pulmonary complications [101]. The efficacy and safety of therapeutic agents, including conventional synthetic disease-modifying anti-rheumatic drugs (csDMARDs) and biologic DMARDs (bDMARDs), in the management of RA-ILD remain subjects of ongoing research and clinical uncertainty [102,103,104]. Nevertheless, growing evidence indicates that these treatments may contribute to slowing disease progression and improving the overall prognosis of RA-ILD [103,105,106,107,108,109,110,111,112,113,114]. Recent studies and meta-analyses have provided accumulating evidence that methotrexate exerts a suppressive effect on ILD [105,115]. The 2021 American College of Rheumatology Guideline for the Treatment of RA conditionally recommend methotrexate over alternative DMARDs for the management of inflammatory arthritis with moderate to severe disease activity in patients with mild, stable airway disease or pulmonary parenchymal disease [58]. Furthermore, in recent years, antifibrotic agents such as nintedanib have demonstrated the ability to slow the decline in forced vital capacity in patients with progressive fibrosing RA-ILD [116]. RA-ILD is a severe organ complication in which early detection is crucial for enhancing patient outcomes. RF titer may prove to be a valuable biomarker for identifying patients at increased risk of developing lung disease or facing mortality [101].

Rheumatoid vasculitis (RV) is a severe extra-articular complication of RA, characterized by inflammation of medium-sized arteries and capillaries. It is associated with a particularly poor prognosis, with high mortality rates attributed to vasculitic damage and the adverse effects of immunosuppressive therapy [117,118]. Several risk factors have been identified for RV, including long-standing RA, male sex, smoking, and the presence of rheumatoid nodules [119,120]. In a study of patients diagnosed with RV between 1980 and 1993, 68% had rheumatoid nodules, and most exhibited strong RF positivity [121]. Furthermore, multiple studies have consistently identified high RF titers as the most significant predictor of RV development [122,123]. Clinically, the prevalence of RV is estimated at 1–5% among RA cases, though autopsy studies suggest a much higher prevalence, ranging from 15 to 31% [124]. This discrepancy underscores the need for vigilant screening to improve early detection and prognosis, further highlighting the critical role of RF in identifying patients at risk for RV.

### 3.4. Difficult-to-Treat RA (D2T RA)

RF is also implicated in the disease mechanism of difficult-to-treat RA (D2T RA), a treatment-refractory subset characterized by inadequate therapeutic responses to ≥2 bDMARDs or targeted synthetic DMARDs with different mechanisms of action [125]. Previous studies have identified various patient-related factors associated with the development of D2T RA, such as young onset, multiple comorbidities, high disease activity, low-dose MTX use, and high-dose glucocorticoid use [126,127,128,129,130,131]. Among them, high RF titers have also been linked to the development of D2T RA [128,129,131], suggesting that high RF titers may serve as a biomarker for identifying patients who would benefit from early aggressive interventions or innovative therapeutic strategies [52,55].

Recent studies identified that RF titers are significantly correlated with serum type I interferon levels, which may contribute to therapeutic resistance in early RA [132,133]. Further investigations are warranted to elucidate the mechanisms by which RF contributes to D2T RA development and to explore its incorporation into personalized treatment paradigms.

### 3.5. RF in the 1987 ACR and 2010 ACR/EULAR Classification Criteria

The evolution of RA classification criteria reflects advancements in our understanding of the disease and the role of serological markers such as RF. The 1987 ACR classification criteria marked a pivotal moment by including RF as one of the key diagnostic parameters [134]. At that time, RF was primarily valued for its diagnostic utility, with its presence supporting a diagnosis of RA in conjunction with clinical symptoms such as joint swelling and morning stiffness. However, the criteria did not consider RF titers, limiting its prognostic utility.

The introduction of the 2010 ACR/EULAR classification criteria represented a significant paradigm shift, emphasizing earlier diagnosis and incorporating both RF and ACPA as critical serological markers [4]. RF’s inclusion was prioritized based on its diagnostic and prognostic value, with higher titers contributing more points toward an RA diagnosis. This quantitative approach not only improved the sensitivity of the criteria but also highlighted the role of RF in identifying patients with more severe disease trajectories. Unlike the 1987 criteria, the 2010 framework acknowledged the additive value of combining RF with ACPA, which together enhance diagnostic specificity and prognostic accuracy.

Importantly, the 2010 criteria also shifted focus toward early RA, aiming to identify and treat patients before significant joint damage occurs. This early intervention model underscores the necessity of RF testing in stratifying patients based on disease severity and progression risk. The integration of RF into both sets of criteria illustrates its enduring relevance in RA classification, while ongoing research into RF isotypes and next-generation assays promises to refine its application further. Future updates to RA classification may benefit from incorporating insights into the differential roles of RF isotypes and their interactions with other biomarkers.

In light of these findings, the prognostic utility of RF in RA extends well beyond traditional diagnostic applications. By integrating RF analysis with other biomarkers and clinical parameters, clinicians can better stratify patients, predict disease trajectories, and tailor treatment plans. Future research should aim to further elucidate the mechanistic pathways linking RF to disease outcomes and explore novel therapeutic approaches targeting RF-mediated processes.

## 4. Therapeutic Implications of RF

The predictive value of RF extends to treatment response. RF-positive patients often exhibit differential responses to DMARDs, including both conventional synthetic and biologic agents. For instance, higher baseline RF titers have been associated with better responses to rituximab, a B-cell-targeting therapy [135], while potentially predicting reduced efficacy of tumor necrosis factor inhibitors [136,137,138,139,140]. This variability highlights the role of RF in guiding personalized treatment strategies and optimizing therapeutic outcomes.

### 4.1. Conventional DMARDs

According to the EULAR recommendations 2022 update for the management of RA, methotrexate (MTX) is recommended as the first-line therapeutic option for patients with RA [52]. MTX, approved by the FDA in 1988, remains the anchor drug and first-line therapy for RA due to its cost-effectiveness and favorable safety profile [141]. Numerous studies have demonstrated the efficacy of MTX as a first-line treatment regardless of the presence of RF or ACPA [142,143,144]. However, some studies suggest that RF positivity may correlate with MTX treatment failure and a higher likelihood of adverse events [145]. Although the precise mechanism underlying the relationship between RF positivity and MTX treatment failure remains unclear, research has shown that type I interferon (IFN) signaling pathway genes are significantly overexpressed in MTX non-responders [146]. Furthermore, the expression of IFN type I regulatory genes is markedly elevated in RA patients compared to healthy individuals and has been positively associated with RF [132,147]. These findings suggest that IFN-related pathways may play a critical role in the link between RF positivity and MTX treatment resistance. Despite these findings, MTX remains the cornerstone of RA management. However, clinical trials evaluating MTX monotherapy have reported that only approximately 40% of patients with early RA achieve a good response as defined by the ACR50 criteria, with even lower response rates observed with csDMARDs other than MTX [148,149,150]. High-titer RF has been identified as a poor prognostic indicator and its presence warrants a more proactive approach, including earlier use of bDMARDs [52].

### 4.2. Tumor Necrosis Factor Inhibitors

Tumor necrosis factor-alpha (TNFα) is a critical mediator that plays a central role in both inflammation and bone destruction in RA [151]. TNF inhibitors—such as infliximab (IFX), etanercept (ETA), adalimumab (ADA), golimumab (GOL), and certolizumab pegol (CZP)—are currently the most widely prescribed bDMARDs due to their proven high efficacy and favorable safety profile [152,153].

**Figure 3:**
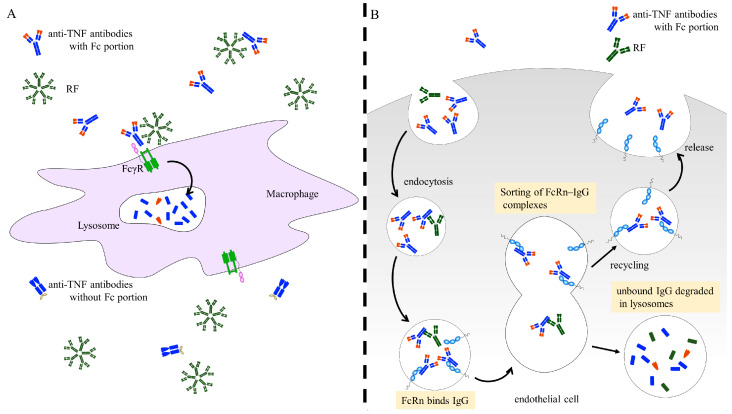
Mechanisms of attenuation of TNF antibody efficacy by RF. (**A**) The RF-IgG immune complex binds to the Fc receptors for IgG (FcγR) on the surface of antigen-presenting cells (APCs), leading to the internalization and subsequent lysosomal transport of the complex. Within the lysosomes, both the FcγR and the internalized antigen undergo proteolytic degradation. Anti-TNF antibodies lacking the Fc portion are hypothesized to evade this degradation pathway. (**B**) The neonatal Fc receptor for IgG (FcRn) is predominantly expressed on endothelial cells. These cells internalize serum IgG and engage FcRn within acidic endosomal compartments. FcRn subsequently recycles IgG back into circulation, thereby extending its serum half-life. In contrast, IgG that fails to bind FcRn is routed to lysosomes for degradation, bypassing intracellular sorting. Rheumatoid factor (RF) binds to the FcRn-binding sites of TNF antibodies and may interfere with their recycling process.

Previous studies have indicated that the presence of RF is associated with a diminished clinical response to TNF inhibitors [136,137,138,139,154,155,156]. However, more recent evidence suggests that CZP, in contrast to monoclonal antibodies such as IFX, ADA, GOL, and the receptor fusion protein ETA, demonstrates consistent efficacy regardless of RF levels [53,157,158,159,160]. The influence of RF on the pharmacokinetics of these therapies represents a critical aspect of our discussion. RFs are autoantibodies that specifically recognize the Fc region of IgG [161]. These RFs form immune complexes with IgG antibodies by binding to their Fc region. These immune complexes are then taken up by antigen-presenting cells (APCs), mainly macrophages, via Fc gamma receptors (FcγRs) for IgG (Figure 3A). Once taken up, these complexes are presumed to be transported to lysosomes, where they undergo enzymatic degradation as part of intracellular processing and clearance mechanisms [162,163]. CZP, however, lacks an Fc region and consequently avoids this pathway [164].

In addition, high-titer RF could impact IgG recycling mediated by the neonatal Fc receptor (FcRn). While TNF inhibitors containing an Fc region are incorporated into the endosome and recycled through binding to FcRn in endothelial cells, IgG that fail to bind to FcRn are degraded in lysosomes [165]. RF may interfere with this recycling process by binding to the FcRn-binding site on IgG [166] (Figure 3B).

Based on these mechanisms, the serum drug levels of TNF inhibitors containing the Fc region, such as IFX and ADA, are reduced or undetectable in patients with high-titer RF. In contrast, the serum drug levels of CZP remain stable regardless of RF titer [159]. Consequently, it has been reported that CZP demonstrates greater efficacy compared to TNF inhibitors with an Fc region in patients with high RF titers (166–204 IU/mL or higher) [53,157,160].

It is important to highlight the recently launched TNF inhibitor ozoralizumab, which is now available in Japan. This next-generation single-domain anti-TNF-α antibody has a unique structure comprising two anti-TNF-α nanobodies^®^ and an anti-human serum albumin (HSA) nanobody^®^. With a molecular weight of 38 kDa—approximately one-fourth the size of conventional anti-TNF-α IgG antibodies—ozoralizumab represents a novel and advanced therapeutic option [167,168]. Notably, ozoralizumab lacks the Fc region in its structure, which may allow it to bypass the efficacy-reducing effects associated with RF.

### 4.3. Non-TNF Targeted Treatment

Treatment with TNF inhibitors have traditionally been the first-line therapy following an inadequate response to MTX. While TNF inhibitors significantly improve the quality of life for most RA patients, at least one-third of patients fail to respond to these drugs [169,170]. To provide alternative options, several newer antirheumatic therapies with distinct mechanisms of action are now available, including non-TNF biologics and Janus kinase (JAK) inhibitors [52,171]. These non-TNF biologics include a range of targeted therapies, including anti-B-cell agents such as rituximab (RTX), anti-T-cell co-stimulatory inhibitors such as abatacept, and interleukin-6 receptor inhibitors such as tocilizumab and sarilumab [172,173,174].

The importance of targeting B cells in the treatment of RA is clear, as evidenced by the efficacy of RTX, a B-cell depletion therapy. A meta-analysis of four randomized placebo-controlled trials (REFLEX, SERENE, DANCER, and IMAGE) revealed that RTX provides additional therapeutic benefits in seropositive RA, particularly in patients who have not responded to at least one TNF inhibitor [135]. Notably, RTX has demonstrated efficacy irrespective of the presence of RF [135]. Furthermore, in a large observational cohort study of RA patients treated with RTX, seropositive patients exhibited significantly lower Disease Activity Score 28 (DAS28) at 6 months compared to seronegative patients [135]. These findings offer critical insights for optimizing treatment strategies in RF-positive patients.

A recent study reported that serum IL-6 concentrations were significantly elevated in patients who failed to achieve remission with TNF inhibitors compared to those who did not. These findings suggest that such patients exhibit a high dependency on IL-6, making IL-6 signaling inhibition a rational and potentially effective therapeutic strategy [175]. While some studies suggest that RF positivity is associated with improved treatment outcomes in patients receiving IL-6 inhibitors, others report no significant correlation between RF positivity and treatment response [176,177,178,179]. Similarly, findings on the relationship between RF presence and treatment response to abatacept have been inconsistent [176,180,181,182,183,184,185], and data regarding RF’s impact on the efficacy of JAK inhibitors remain limited [186,187,188]. However, there is no evidence to indicate that the presence of RF is linked to treatment discontinuation due to a lack of efficacy for these therapies. Consequently, the presence or absence of RF does not seem to restrict the clinical application of these therapies.

## 5. Conclusions

Over the decades, advancements in RF detection methods and a deeper understanding of its isotype-specific roles have significantly improved RA management. However, challenges such as diagnostic specificity, prognostic variability, and treatment resistance persist, highlighting the need for further research. Future efforts should focus on refining RF assays to enhance their diagnostic and prognostic precision, exploring the temporal dynamics of RF isotype progression, and integrating RF into personalized treatment paradigms.

Given the heterogeneity of RA, RF remains a key biomarker not only for its established diagnostic and prognostic value but also for its emerging role in guiding treatment stratification. RF, in combination with other biomarkers such as ACPA, may help identify patient subgroups more likely to benefit from specific therapeutic approaches. Moreover, high RF titers have been linked to treatment resistance, particularly with MTX and certain biologic therapies, underscoring the importance of personalized treatment strategies. Integrating RF status into precision medicine frameworks holds promise for optimizing therapeutic outcomes and advancing individualized RA management.

## Data Availability

Not applicable.
